# Supplementary material for: Antitumor Effect of Bleomycin Nanoaerosol in Murine Carcinoma Model
Source: Molecules. 2023 May 18;28(10):4157. doi: 10.3390/molecules28104157 (PMC10221970; doi:10.3390/molecules28104157)
Supplement: Supplementary file 1 [file molecules-28-04157-s001.zip › molecules-2356951-supplementary.pdf]

Supplementary Materials

Bleomycin nanoaerosol treatment induces inhibition of pulmonary metastases in murine carcinoma model

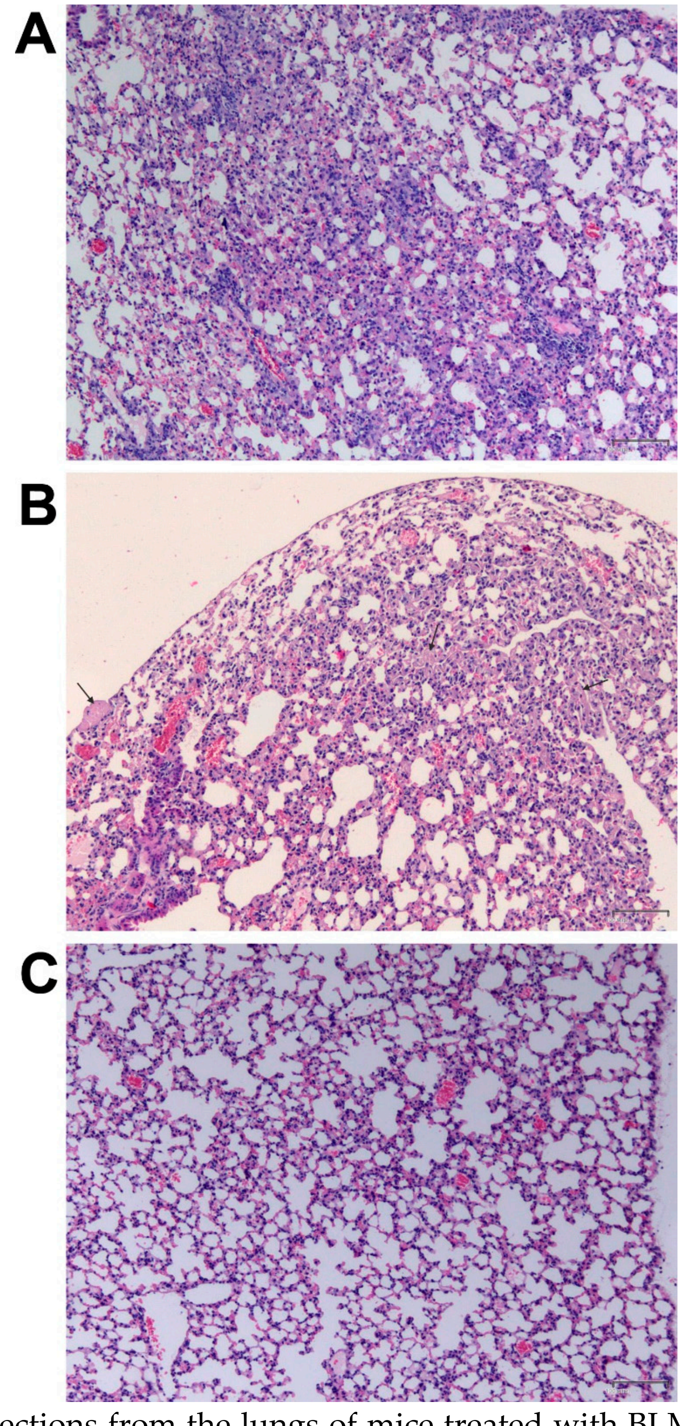

Figure S1. Representative sections from the lungs of mice treated with BLM NAPs (A), treated with intratracheal BLM injection (B) and untreated animal (C). Arrows point to areas with pneumofibrosis (B).

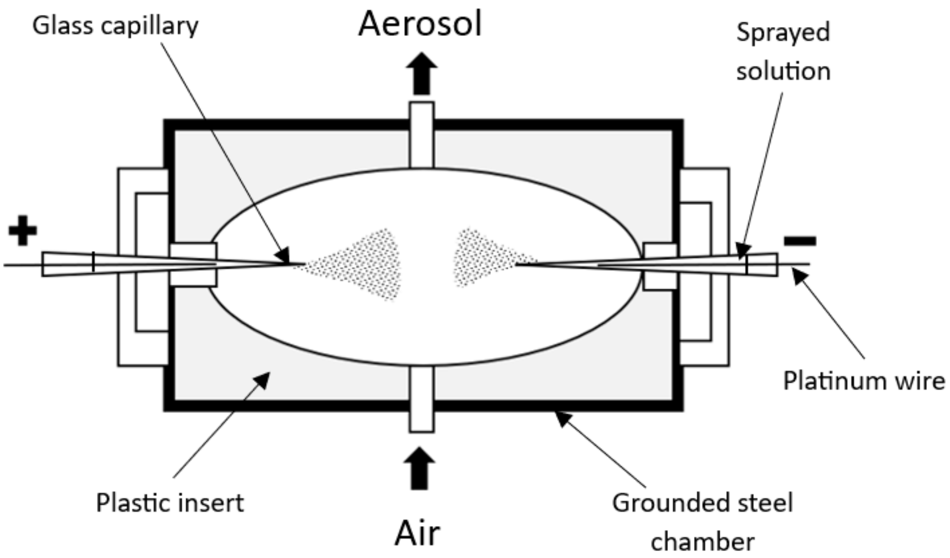

Figure S2. A schematic illustration of the electrospray-based nanoaerosol generator.

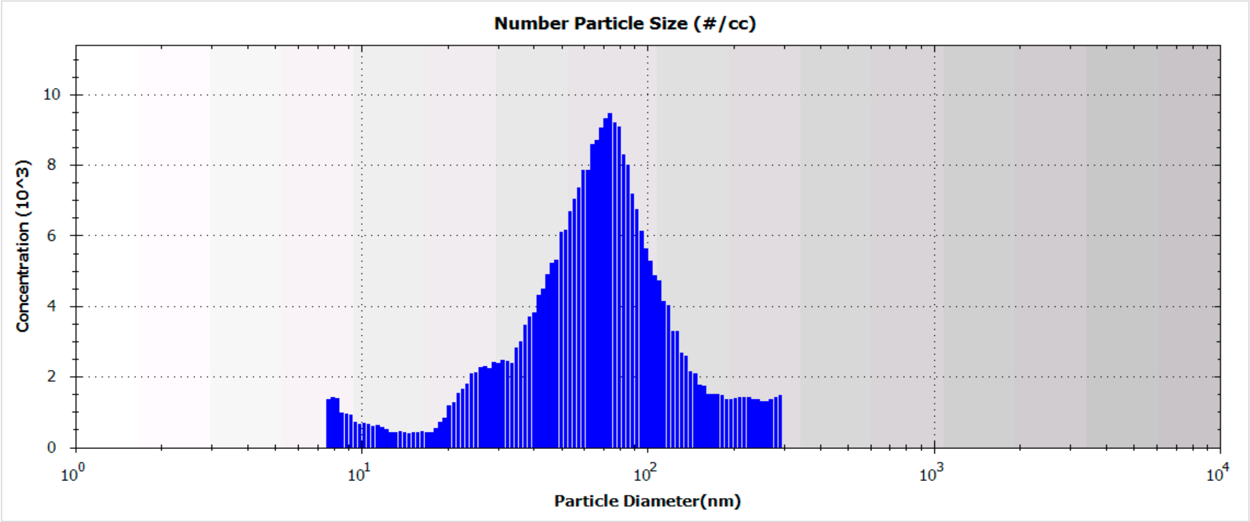

Figure S3. Size distribution of glucose NAPs in aerosol generated by spraying of 1% solution of glucose in 20% ethanol.

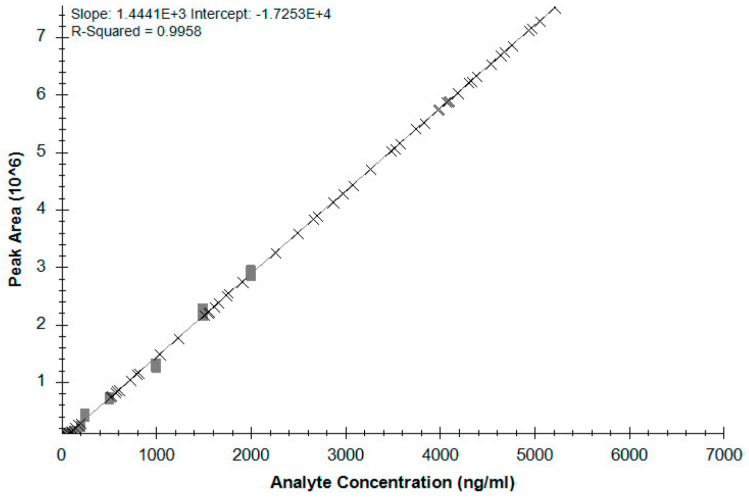

Figure S4. The calibration curve used for LC-MS-based estimation of BLM concentration in mouse plasma. Grey squares depict experimental points.

Table S1. Daily aerosol concentration measurements and expected inhaled doses of bleomycin in experiment with **B16F10 melanoma** murine model (**experiment 1**, corresponds to group 1 in **Antitumor effect of NAPs on murine melanoma model** section, inhalation 5 h/day).

**Day**	**Aerosol bleomycin concentration (c), μg/L**	**Daily dose, μg/mouse**
1	0,27	0,73
2	0,33	0,88
3	0,22	0,58
4	0,19	0,51
5	0,11	0,29
6	0,19	0,51
7	0,49	1,31
8	0,14	0,36
9	0,22	0,58
10	0,22	0,58
11	0,19	0,51
12	0,03	0,07
13	0,22	0,58
14	0,36	0,95

Table S2. Daily aerosol concentration measurements and expected inhaled doses of bleomycin in experiment with **B16F10 melanoma** murine model (**experiment 3**, corresponds to group 2 in **Comparative effectiveness of NAPs and injections of bleomycin** section, inhalation 2.5 h/day)

**Day**	**Aerosol bleomycin concentration (c), µg/L**	**Daily dose, µg/mouse**
1	0,52	0,69
2	0,52	0,69
3	0,52	0,69
4	0,35	0,46
5	0,35	0,47
6	0,35	0,47
7	0,02	0,02
8	0,02	0,03
9	0,02	0,03
10	0,32	0,42
11	0,32	0,43
12	0,32	0,43
13	0,36	0,47
14	0,36	0,48

Table S3. Daily aerosol concentration measurements and expected inhaled doses of bleomycin in experiment with B16F10 melanoma murine model (**experiment 2**, corresponds to group 1 in **Comparative effectiveness of NAPs and injections of bleomycin** section, inhalation 5 h/day).

**Day**	**Aerosol bleomycin concentration, c, µg/L**	**Daily dose, µg/mouse**
1	0,26	0,69
2	0,31	0,83
3	0,33	0,87
4	0,35	0,94
5	0,32	0,86
6	0,16	0,42
7	0,01	0,03
8	0,02	0,04
9	0,02	0,04
10	0,07	0,18
11	0,18	0,47
12	0,17	0,45
13	0,18	0,49
14	0,31	0,82

Table S4. Daily aerosol concentration measurements and expected inhaled doses of bleomycin experiment with Lewis lung carcinoma murine model (corresponds to group 1 in **Antitumor effect of NAPs on murine Lewis lung carcinoma model** section, inhalation 5 h/day).

Day	Aerosol bleomycin concentration, μg /L	Daily dose, μg /mouse
1	0,17	0,46
2	0,17	0,46
3	0,69	1,85
4	0,69	1,85
5	0,60	1,58
6	0,60	1,58
7	0,43	1,15
8	0,43	1,15
9	0,49	1,31
10	0,49	1,31
11	0,46	1,23
12	0,46	1,23
13	0,14	0,37
14	0,14	0,37

Table S5. Relative weights of spleen after i.p. and aerosol administration of BLM and in control group. Data are mean of ten mice per group.

Groups	Relative weight of spleen, %
Control	0.75
BLM NAPs 5 h	0.76
BLM NAPs 2.5 h	0.64
BLM i.p. 8 mg/kg	0.44
BLM i.p. 4 mg/kg	0.42
